# Effect of hydrogen peroxide versus charcoal-based whitening mouthwashes on color, surface roughness, and color stability of enamel

**DOI:** 10.1186/s12903-024-04631-w

**Published:** 2024-08-06

**Authors:** Mayada S. Sultan

**Affiliations:** Operative Dentistry Department, Faculty of Dentistry, Fayoum University, Fayoum, Egypt

**Keywords:** Hydrogen peroxide, Charcoal, Whitening mouthwashes, Color change, Surface roughness, Color Stability

## Abstract

**Background:**

Patients tend to favor the whitening mouthwashes as they are easily applied and affordable. This study aimed to evaluate the effect of hydrogen peroxide versus charcoal-based whitening mouthwashes on color, surface roughness, and color stability of enamel. In the current study, the whitening mouthwashes used have the ability to stop future stains due to their white seal technology.

**Methods:**

A total of 21 permanent central incisor teeth extracted for periodontal reasons were used in the present study. Teeth roots were sectioned and crowns were mounted in self-cured acrylic resin blocks. The specimens were randomly divided into three groups (n = 7) according to the tested whitening mouthwash: Control group ‟ DW” (Distilled water), ‟OW” group: Peroxide-based mouthwash (Colgate Optic White) and ‟CP” group: Charcoal-based mouthwash (Colgate^®^ Plax Charcoal). Regarding ‟OW” and ‟CP” groups, the specimens were immersed in 20 ml of the tested mouthwash in each corresponding group for 1 min twice daily (morning and evening) for a total of 12 uninterrupted weeks. Color change was assessed using VITA Easyshade spectrophotometer and surface roughness (Ra) was measured using a white light interferometer. The specimens were stained using black tea solution and color was measured after 24 h of immersion for assessment of color stability.

**Results:**

Color change results revealed that both whitening mouthwashes were able to restore color comparable to the control group with no significant difference between them. Regarding surface roughness, the control group showed the highest mean Ra value, followed by ‟OW” group while ‟CP” group showed the lowest mean Ra value. While color stability after staining, the control group showed a significantly higher value than the ‟CP” and ‟OW” groups.

**Conclusion:**

Hydrogen peroxide and charcoal-based whitening mouthwashes improve the color of enamel with no adverse effect on the surface roughness. Both whitening mouthwashes were beneficial to maintain the color after staining and prevent future enamel stains.

## Introduction

In current society, the color of the teeth is one of the most significant variables impacting the esthetic harmony of the smile because it is often observed before many other esthetic defects [1]. Teeth discoloration can be caused by intrinsic or extrinsic factors. Extrinsic stains typically result from smoking, bad dental hygiene, or foods with high chromophores content such as coffee and tea [2]. Particularly when there are rough tooth surface present, organic and inorganic chromophores are immediately adsorbed onto this surface [3]. Thus, for patients seeking cosmetic dentistry, teeth whitening has become a necessary procedure to improve tooth color [4].

Tooth bleaching remains one of the most prevalent conservative procedures for treating the discolored natural teeth [5]. The literature has described a variety of teeth-whitening techniques, including at-home, in-office, and over-the-counter bleaching products [6]. Patients tend to favor over‑the‑counter whitening products as they are easily applied, affordable, and readily available at pharmacies, supermarkets, and online shopping [7]. Examples of such products include mouthwashes, strips, toothpaste, whitening paint-on products, and chewing gum [8].

Mouthwashes are used as oral hygienic aids to prevent halitosis. They contain a combination of antimicrobials, water, salts, coloring agents, and sometimes alcohol [7]. The formulation of whitening mouth washes typically contains low levels of hydrogen peroxide, ranging from 1.5 to 6% [1]. The action of hydrogen peroxide is based on the release of reactive oxygen particles, which diffuse through the enamel surface and oxidizes the chromogens’ double bonds, breaking them into smaller ones, resulting in a lighter colored tooth surface [9, 10].

Currently, whitening mouth rinses are no longer based on hydrogen peroxide only, as activated charcoal has been introduced to whitening products and has become a widely used stain removal product [11]. Basically, charcoal is a carbon-rich material that is compacted with high porosity as a result of burning organic matter such as wood, nutshell, bamboo, and coconut husk. It has the power to adsorb gases, impurities, and liquids inside its pores [12]. Consequently, the high porosity and large specific surface area resulted from the nanocrystalline form of carbon that has the ability to purify, deodorize, clarify liquids and gases [13].

Nowadays, it is important to develop whitening mouthwash to not only remove the stains but also prevent future stain formation. The manufacturer claims that Colgate^®^ Plax Charcoal (charcoal-based) mouthwash and Colgate Optic White Advanced (peroxide-based) mouthwash have the ability to stop future stains due to their white seal technology, which prevents future stains.

One of the undesired effect of whitening agents is the increasing surface roughness that is directly related to tooth discoloration [14]. Although whitening agents is positively affect tooth color, they could negatively affect surface roughness of enamel [15]. The new mouthwash formulations with white seal technology supposed to maintain enamel color stability and have minimal effect on its surface roughness. Therefore, this study was conducted to evaluate the effect of hydrogen peroxide versus charcoal- based whitening mouthwashes on color, surface roughness, and color stability of enamel.

## Materials and methods

### The sample size calculation

A power analysis was designed to have adequate power applying a statistical test of the null hypothesis that there is no difference between different tested groups regarding color change. By adopting an alpha (α) and beta (β) levels of (0.05), (i.e., power = 95%) and an effect size (f) of (0.946) calculated based on the results of a previous study by Favaro et al. [16] of the minimum total required sample size (n) was found to be (21) samples (i.e., 7 samples per group). Sample size calculation was performed using R statistical analysis software version 4.3.2 for Windows^1^.

### Teeth selection and specimens’ preparation

Twenty-one human permanent central incisor teeth extracted for periodontal reasons were used in the present study. The study was approved by Scientific Research Ethical Committee, Faculty of Medicine, Fayoum University with a research code (R 513 - session 112 − 12/11/2023). The teeth were washed under running water, scaled from adhering soft tissue and plaque, and then stored at 4ºC in distilled water for not more than one month. Teeth roots were cut 2 mm below the cemento-enamel junction. The coronal portions were mounted in self-cured acrylic resin blocks using metal molds (2 cm x 3 cm) with the labial surface facing upward. Enamel was wet-ground using 80 grit sandpaper discs to achieve flat enamel surfaces. Enamel surfaces were abraded with 400 and 600 grit sandpaper discs and polished with rubber cups and paste to achieve smooth surfaces. After polishing, the specimens were cleaned in an ultrasonic cleaning device (Wisd, WUC-D06H, DAIHAN Scientific Co, Ltd, Korea) with deionized water for 15 min to remove any debris. All the specimens were stored in distilled water, which was changed daily until the testing procedures and specimen grouping.

### Specimens’ grouping

The specimens were randomly divided into three groups (n = 7) according to the tested whitening mouthwash as follows: Control group ‟DW” (Distilled water), ‟OW” group: Peroxide-based mouthwash (Colgate Optic White) and ‟CP” group: Charcoal-based mouthwash (Colgate^®^ Plax Charcoal). Table 1 shows the trade name, description, ingredients, manufacturer, and lot number of the whitening mouthwashes used in the current study.

**Table 1:** Whitening mouthwash used in the current study

Material Trade name and Description	Ingredients	Manufacturer and lot No.
**Colgate Optic White** (Advanced whitening mouthwash 2% hydrogen peroxide with whiteseal technology and alcohol-free )	Hydrogen Peroxide, Polysorbate 20, Sodium Acrylates/Methacryloylethyl Phosphate Copolymer, Water, Glycerin, Propylene Glycol, Sorbitol, Phosphoric Acid, Citric Acid, Flavor, PVM/MA Copolymer, Sodium Saccharin.	01082006 Colgate Palmolive manufacturing Poland SP. Z O.O. Colgate 2 street, 58–100 Swidnica, Poland
Colgate^®^ Plax Charcoal (Charcoal-based whitening mouthwash with whiteseal technology and alcohol-free)	Charcoal Powder, Polysorbate 20, Tetrapotassium Pyrophosphate, Tetrasodium Pyrophosphate, Zinc Citrate, PVM/MA Copolymer, Aqua, Glycerin, Propylene Glycol, Sorbitol, Aroma, Benzyl Alcohol, Sodium Fluoride, Sodium Saccharin, Bambusa Vulgaris Shoot Extract, CI 15,510, CI 17,200, CI 19,140, CI 42,051.	10,197,702 Colgate Palmolive manufacturing Poland SP. Z O.O. Colgate 2 street, 58–100 Swidnica, Poland

### Mouthwash simulation

Regarding control group ‟DW” the specimens were immersed in distilled water, which was changed daily. On the other hand, ‟OW” group: Peroxide-based mouthwash (Colgate Optic White) and ‟CP” group: Charcoal-based mouthwash (Colgate^®^ Plax Charcoal), the specimens were immersed in 20 ml of the tested mouthwash in each corresponding group for 1 min twice daily (morning and evening) for a total of 12 uninterrupted weeks, simulating clinical application, as indicated by manufacturers. After immersion, the specimens were washed with distilled water and then stored in distilled water until use to avoid dehydration.

### Color assessment

The colors of the specimens were measured three times: at baseline, after whitening mouthwash application, and after staining. Color of the specimens was assessed before any treatment (baseline) according to CIE (Lab) color system (Commission Internationale de L’Eclairage) by using a VITA Easyshade spectrophotometer (Advance 4.01, VITA Zahnfabric, Bad Sackingen, Germany) against a white background. According to this system, the three different color parameters L*, a* and b* were calculated as follow: L* value denotes darkness–brightness (range from 0 to 100); a* value represents the green–red component (ranging from–80 green to + 80 red) and b* represents the blue–yellow component (values ranging from − 80 blue to + 80 yellow). Color of the middle portion of each specimen was recorded [17]. The color changes were further assessed after the use of mouth washes then after staining and color change (∆E) was then calculated according to the following formula CIEDE2000 (ΔE00): [18].

$$\begin{aligned}\Delta E_{{\text{00}}} &= \surd( ((\Delta L^{\prime})/(K_L S_L ))^2+ ((\Delta C^{\prime})/( K_C S_C ))^2\\&\quad+((\Delta H^{\prime})/( K_H S_H ))^2+R_T  (\Delta C^{\prime})/( K_C S_C )  (\Delta H^{\prime})/(K_H S_H )  )\end{aligned}$$

where ΔL′, ΔC′, and ΔH′ denote lightness, chroma, and hue differences between color measurements. K_L_, K_C_, and K_H_ denote the parametric factors to be adjusted according to different viewing parameters [8, 9]. S_L_, S_C_, and S_H_ denote the weighting functions for the adjustment of color difference considering the location variation of L*, a*, and b* color coordinates. RT denotes the function for the hue and chroma differences interaction in the blue region [8].

### Surface roughness assessment

Surface roughness was measured using a white light interferometer at baseline and after application of whitening mouthwash. This was carried out using the ZYGO Maxim-GP 200 profilometer (Laurel Brook Rd, Middlefield, CT 06455, United States), which is a general-purpose surface optical profiler that measures the microstructure and topography of surfaces in three dimensions. Computerized phase stepping interferometry (PSI) upgraded with scanning white light interferometry (SWLI) and advanced surface texture software was used, which analyzes areas as well as profiles and step height. A white light from a halogen lamp incident on an interference filter with Full Width at Half Maximum (FWHM) ≈ 3–15 nm was used depending on the measuring technique. Three readings were recorded for each specimen, and an average value was calculated to represent the surface roughness for each specimen in µm.

### Staining of the specimens

After 12 weeks of whitening procedures the specimens of the ‟OW” and ‟CP” groups, in addition to the specimens of the control group ‟DW”, were stained using a black tea solution (Yellow Label, Lipton black tea, made in Kenya, imported and packaged in Egypt, New Borg El Arab, Alexandria). The solution was prepared by immersing two tea bags (2 × 2.0 g) into 200 mL of boiling water for 3 min, then filtering with a piece of gauze. The specimens were immersed in the tea solution for 24 h (long exposure), according to Palandi et al., [5] which simulated one-month consumption. After that, the specimens were rinsed, stored in distilled water, and then dried after storage to reevaluate their color.

### Statistical analysis

Numerical data were presented as mean and standard deviation (SD) values. Normality and variance homogeneity assumptions were confirmed by viewing the distribution and by using Shapiro-Wilk’s and Levene’s tests respectively. Intergroup comparisons were analyzed using one-way ANOVA followed by Tukey’s post hoc test. The significance level was set at *p* < 0.05 within all tests. Statistical analysis was performed with R statistical analysis software version 4.3.2 for Windows^2^.

## Results

**Fig. 1:**
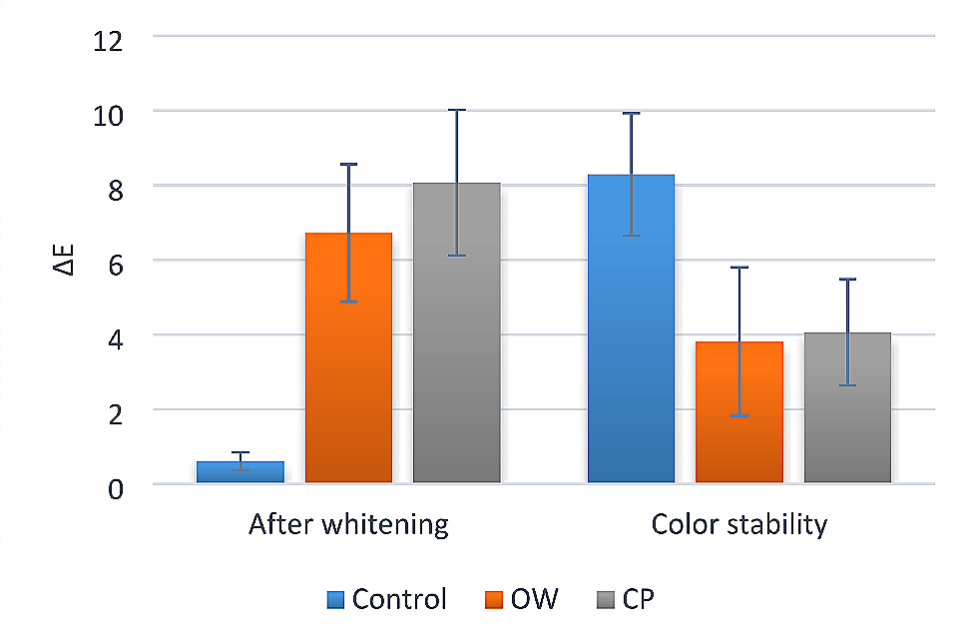
Bar chart showing the color change of ‟OW” and ‟CP” groups after whitening and the color stability of three tested groups after staining

Intergroup, intragroup comparisons and summary of statistics for color change (ΔE) are presented in Table 2; Fig. 1. For both deltas (after application of whitening mouthwashes and after staining), there was a significant difference between the tested groups (*p* < 0.001). After application of whitening mouthwashes, post hoc pairwise comparisons showed the OW group and the CP group had significantly higher values than the control group (*p* < 0.001). While color stability after staining, the control group showed a significantly higher value than the CP and OW groups (*p* < 0.001).

**Fig. 2:**
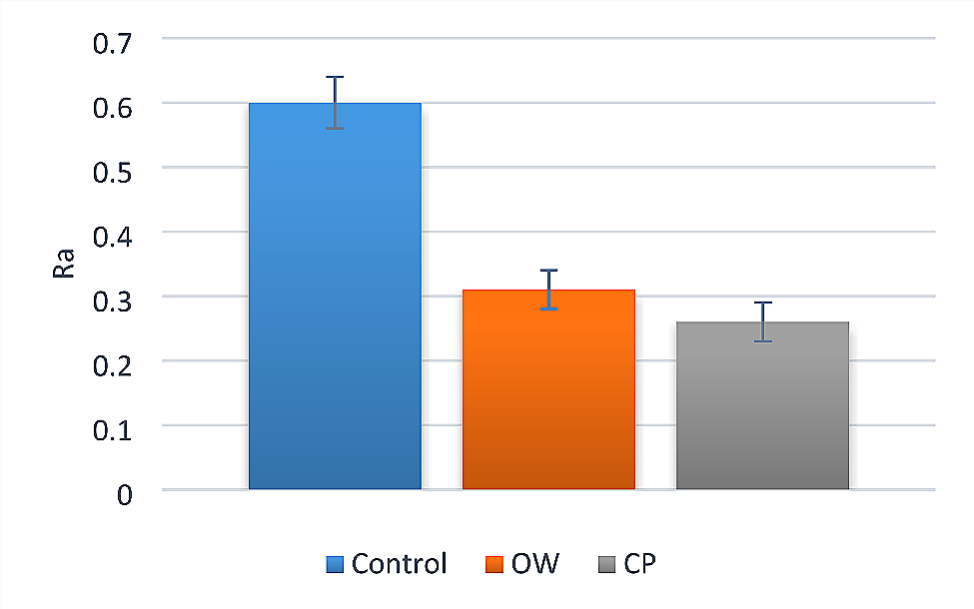
Bar chart showing the surface roughness of the three tested groups

**Fig. 3:**
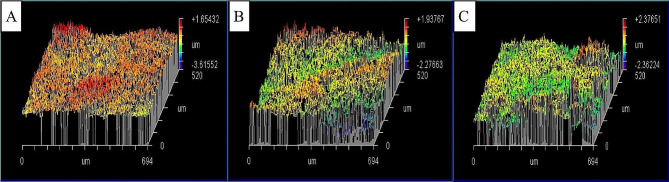
Interferometer images of enamel surface roughness for the three tested groups; A: Control group, B: Peroxide-based mouthwash and C: Charcoal-based mouthwash

Intergroup comparisons and summary statistics for surface roughness (Ra) are presented in Table 3; Fig. 2. Results showed that the difference between tested groups was statistically significant (*p* < 0.001). Post hoc pairwise comparisons revealed that there was a statistically significant difference between the tested groups (*p* < 0.001). The control group showed the highest mean Ra value, followed by OW group while CP group showed the lowest mean Ra value. Figure 3 showed representative interferometer images of enamel surface roughness for the three tested groups.

**Table 2:** Inter, intragroup comparisons and summary statistics for color change (ΔE)

Measurement	Color change (ΔE) (Mean ± SD)			Test statistic	*p*-value
	Control	OW	CP		
**After whitening**	0.61 ± 0.24^B^	6.72 ± 1.84^A^	8.07 ± 1.95^A^	**45.75**	< 0.001*
**Color stability**	8.29 ± 1.64^A^	3.81 ± 1.99^B^	4.06 ± 1.42^B^	**15.37**	< 0.001*

Means with different superscript letters within the same horizontal row are significantly different; *significant (*p* < 0.05)

**Table 3:** Intergroup comparisons and summary statistics for surface roughness (Ra)

Surface roughness (Ra) (Mean ± SD)			Test statistic	*p*-value
Control	OW	CP		
0.60 ± 0.04^A^	0.31 ± 0.03^B^	0.26 ± 0.03^C^	**216.79**	< 0.001*

Means with different superscript letters within the same horizontal row are significantly different; *significant (*p* < 0.05)

## Discussion

The current study was carried out to evaluate the effect of hydrogen peroxide (Colgate Optic White Advanced) versus charcoal - based (Colgate^®^ Plax Charcoal) whitening mouthwashes on color, surface roughness, and color stability of enamel.

The main attraction of over-the-counter (OTC) whitening products is that they can be easily purchased and applied independently by patients without dentist’s supervision [1]. This type of product doesn’t require a prescription or professional application and is available in various presentations, such as whitening dentifrices, mouth rinses, chewing gum, and paint-on films [7]. They are used as oral hygiene auxiliaries, for halitosis prevention, and for teeth bleaching because their formulations contain low concentrations of a bleaching agent [7, 16].

Currently, the use of whitening mouthwashes has risen due to increased patient concern about dental esthetics. The decision to use such products is entirely patient-dependent, without any diagnosis for the discoloration causes [20]. The complication due to the prolonged use of whitening mouth rinses may negatively affect the enamel surface. Previous studies [21, 22] revealed that the vulnerability of enamel to staining after whitening procedures was in direct relation to the surface roughness. Since the rough enamel surfaces are more susceptible to stains, the colored food could stick more significantly to them [21].

Results of color change after the application of whitening mouthwashes revealed that ΔE showed a significant difference between the two tested mouthwashes. ‟OW” group and ‟CP” group have significantly higher values than the control group ‟DW” (*p* < 0.001). Colgate Optic white advanced whitening mouthwash contains a low concentration (2%) of hydrogen peroxide that is able to whiten teeth through the release of reactive oxygen molecules. These low-molecular-weight molecules diffuse through the inter-prismatic spaces, breaking long-chain, dark-colored complex chromophore molecules into smaller ones and providing the desired color change that leads to successful whitening [8, 23]. This finding agreed with other studies [24, 25], which demonstrated that low concentrations of peroxide are able to produce a more noticeable and acceptable color change. However, this result was contradicted by a previous study [7] that reported that low concentrations of hydrogen peroxide in bleaching agents could limit their clinical effectiveness.

On the other hand, the whitening effect of Colgate Plax charcoal-based whitening mouthwash is attributed to the presence of charcoal, which has become popular in oral hygiene products aiming to improve the removal of extrinsic stains and achieve tooth bleaching [13]. Charcoal is able to bind to the tooth surface, absorb the dark chromophores in its pores, and provide whitening action [11]. Therefore, the use of Colgate Plax charcoal-based whitening mouthwash in the present study was able to produce a significant whitening effect on enamel because of this charcoal ability. However, this result disagreed with previous study [26] that revealed the use of charcoal-containing mouthwash did not improve the color. This difference may be related to differences in mouthwash composition and application method.

Surface roughness (Ra) results revealed that there was a statistically significant difference between the tested groups (p < 0.001). The control group showed the highest mean Ra value, followed by the ‟OW” group, while the ‟CP” group showed the lowest mean Ra value. The control group in the present study was distilled water, and all the specimens from the other groups were kept in distilled water between immersion periods. The use of distilled water as storage media and control group in the current study is attributed to eliminate of the possible potential remineralizing effect that might be formed when using the artificial saliva. As the precipitation of minerals form artificial saliva on enamel surface may affect the surface roughness results and cause remineralization [27].

The ‟OW” group showed a lower mean Ra value in comparison to the control group. The low enamel surface roughness may be attributed to a low hydrogen peroxide percentage of 2% within the Colgate Optic white whitening mouthwash used in this study. In addition, this mouthwash is alcohol-free, and has a pH value of about 7 [28], which may be helpful to whiten enamel without damaging the surface roughness. This result was in accordance with Yildirim et al., [24], but it disagreed with a previous study [7] that revealed enamel suffered damage and surface roughness changes with low-peroxide-content mouthwash. This difference may be attributed to multiple factors, including the mouthwash composition and pH value.

On the other hand, the ‟CP” group showed the lowest mean Ra value. This might be explained by the whitening mouthwash content of sodium fluoride, which helps in enamel remineralization. Moreover, the Colgate Plax whitening mouthwash used in the present study is alcohol-free, and has a pH value of about 7.9 [29] that provide safe enamel whitening. This finding was in accordance with Dionysopoulos et al., 2020 [26], who reported that mouthwash containing charcoal has no morphological alterations on the enamel surface. However, it disagreed with a previous study [11] that revealed an increase in enamel surface roughness using whitening toothpaste with activated charcoal. This difference may be attributed to presentation of charcoal, as this study used it in toothpaste form, brushing technique, and the study design.

Extrinsic discoloration is caused mainly by the adsorption of polyphenolic compounds onto the enamel surface and their interaction with pellicle proteins [30]. Most of these organic chromogens are present in food and beverages. However, the extrinsic staining deposited on the enamel surface can be removed with over-the-counter whitening products [5]. Moreover, the enamel surface is more susceptible to staining after bleaching and whitening procedures due to surface roughness and the imperfections resulting from the bleaching process [21].

In the present study, enamel staining was done using black tea for 24 h. Previous studies reported artificial tooth staining with coffee [30], soft drinks [31], black tea [32], and red wine [33]. There is an evidence that black tea exhibits a higher staining effect than other coloring beverages [34]. In this regard, Sulieman et al. (2003) [35] validated an artificial staining protocol with black tea for teeth bleaching evaluation. According to the authors, the overnight staining protocol would (24-hour immersion) of enamel and dentin in black tea solution did not differ from a 6-day immersion.

The results of color stability after staining with tea revealed that both the ‟OW” and the ‟CP” whitening mouthwashes were able to inhibit stain formation. According to the manufacturer, the white seal technology the whitening mouthwashes used in this study prevents future stains. This may be attributed to mouthwash ingredients such as the presence of acrylates/methacryloylethyl phosphate copolymer within Colgate Optic White and tetrapotassium pyrophosphate (which is a white hydroscopic powder) and tetrasodium pyrophosphate in Colgate^®^ Plax whitening. These materials act as stain prevention actives, which are able to chemically remove the existing stains and help to protect the enamel surface from further stain buildup, providing long-lasting inhibition of new-stain chromogen adsorption to the tooth surface [8]. The results of color stability after staining for the ‟OW” and ‟CP” groups may correlate to the results of low surface roughness for both groups. There are no available studies in the literature about the effect of whitening mouthwashes on color stability of enamel using the whitening mouthwashes that were used in this study with the new white seal technology that whitens teeth and prevents future stain formation; therefore, this result cannot be directly compared.

### Study limitations

It is hard to simulate the complex oral environment. The in-vitro design of this study is considered to be a limitation of this study. In addition, color stability depends on the consumption of staining beverages and food, which could vary according to personal preference and is hard to be unified. Further in-vivo studies are needed to evaluate the color stability of human enamel after using the whitening mouthwashes available on the market.

## Conclusions

Considering the limitations of the present in vitro study, it could be concluded that hydrogen peroxide and charcoal-based whitening mouthwashes improve the color of enamel with no adverse effect on the surface roughness. Both whitening mouthwashes were beneficial to maintain the color after staining and prevent future enamel stains.

## Data Availability

Available on request from the corresponding author, Dr. Sultan M.

## Footnotes

1 R Core Team (2023). R: A language and environment for statistical computing. R Foundation for Statistical Computing, Vienna, Austria. URL https://www.R-project.org/.

2 R Core Team (2024). R: A language and environment for statistical computing. R Foundation for Statistical Computing, Vienna, Austria. URL https://www.R-project.org/.
